# Biased Opioid Ligands: Revolution or Evolution?

**DOI:** 10.3389/fpain.2021.722820

**Published:** 2021-09-24

**Authors:** Florence Noble, Nicolas Marie

**Affiliations:** Université de Paris, CNRS ERL 3649 Pharmacologie et thérapies des addictions, Inserm UMR-S 1124 T3S Environmental Toxicity, Therapeutic Targets Cellular Signaling and Biomarkers, Paris, France

**Keywords:** opioids, biased ligands, analgesia, side effects, TRV130, PZM21

## Abstract

Opioid are the most powerful analgesics ever but their use is still limited by deleterious side effects such as tolerance, dependence, and respiratory depression that could eventually lead to a fatal overdose. The opioid crisis, mainly occurring in north America, stimulates research on finding new opioid ligands with reduced side effects. Among them, biased ligands are likely the most promising compounds. We will review some of the latest discovered biased opioid ligands and see if they were able to fulfill these expectations.

## Introduction

Opium consumption and its medicinal application dates back to the Neolithic, Bronze, and Iron Age. Ancient Egyptian papyrus records reported the use of opium for pain relief, demonstrating that opioids are used from thousands of years for the treatment of pain. Opium is a substance extracted from *Papaver somniferum*, and opium poppies produce alkaloids such as morphine (1). In 1973, the notion of “morphine receptors” emerged (2), and since that time numerous studies have been performed on these receptors. In the 1990s, opioid receptors were cloned. They are four of them, MOR, DOR, KOR, and NOR, and are largely found within the central nervous system as well as throughout the peripheral tissues. They are naturally stimulated by endogenous peptides (endorphins, enkephalins, dynorphins, and nociceptin/orphanin FQ). All pharmacological studies confirmed that opioid system plays a key role in the modulation of pain perception, but is also involved in many physiological responses, such as respiration, gastrointestinal motility, endocrine, and immune functions, which may lead in clinical practice to adverse effects when pain must be managed with opioids.

The study of responses of opioid receptors-deficient mice to opioid agonists clarifies the biological activity of each receptor. The genetic approach unambiguously demonstrated that MOR are essential for all the biological activities elicited by morphine, including for instance analgesia, reward, respiratory depression, constipation, immunosuppression (3). Thus, a challenge over the past decades has been to try to find MOR opioid ligands able to induce a potent analgesic response, with fewer adverse effects.

MOR belong to the G protein-coupled receptors (GPCR), and it is now well-established that once the receptor is activated, several intracellular signaling pathways may be activated. Among these pathways, the focus has been made on two of them, G protein (and its subsequent effectors such as adenylate cyclase and ion channels) and beta-arrestin, and it has been suggested that analgesic effects of MOR ligands are mediated by the downstream G-protein-dependent pathway, while beta-arrestin-dependent pathway mediates most of the side effects. Thus, it was speculated that development of biased MOR ligands for G-protein-dependent pathway may have high analgesic potency, but with fewer undesirable effects. The main goal of this review is to briefly describe this strategy and to discuss its advantages and limits.

## Side Effects of Opioids and Interest in Biased Agonism

With great therapeutic potential, usually comes great side effects. The opiates do not seem to deviate from this rule. Opioid receptors and especially MOR are highly expressed in the brain structures involved in control of breathing including the pre-Bötzinger complex or the Kolliker-Fuse neurons (4). Activation of MOR in the pre-Bötzinger complex with fentanyl promotes a respiratory depression (5) and the knockout of MOR in this complex reduced the effect of morphine on breathing (6). MOR are also expressed in the enteric nervous system and their activation will cause reduction of gastrointestinal motility and thus constipation (7).

Following a protracted exposure, but also after an acute exposure at high dose, a tolerance to opioid-induced pharmacological effects appears. It noteworthy that the level of tolerance depends on the pharmacological effect studied. For instance, there is evidence to suggest that the tolerance to respiratory depression is weak as compared to the antinociceptive tolerance (8) which might explained the fatal overdose that could occur in opioid users (9). Finally, a repeated use of opioids, even in the frame of a pain treatment, could lead to development of addictive behaviors. This is well-illustrated by the opioid crisis in US where postoperative opioid prescription is a significant contributor to opioid epidemic, and more than 350,000 people died from opioid overdose between 1999 and 2016 (https://www.cdc.gov/drugoverdose/epidemic/index.html). All these side effects are a real impairment in the opioid use, particularly in chronic pain. Among the different strategies to find potent analgesics with limited side effects, the one based on the particular properties of the opioid receptors and their ligands, the biased signaling, has received great attention since few years even though the concept (as known as functional selectivity) is older (10). Indeed, the notion of functional selectivity was probably first suggested in a review on serotoninergic receptors where the ability of a single GPCR to active one or more distinct signaling cascades was described (11). And the term of biased agonist was first introduced by Jarpe and co-workers when they observed that a peptide promoted a biased activity on chemokine receptors (12). For opioid receptors, the studies of Bohn and co-workers along with this notion opened the way to find new opioid ligands. As rapidly mentioned earlier, following ligand binding, MOR activation could result in the activation of multiple downstream pathways through either G protein dependent processes (e.g., adenylate cyclase inhibition or regulation of ion channels) or G protein independent processes (e.g., beta-arrestin signaling). Beta-arrestins are proteins, existing in two isoforms (beta-arrestin 1 and 2), that bind the activated and phosphorylated receptor and are responsible for its desensitization and internalization (13). In beta-arrestin 2 knockout mice, Bohn and collaborators found that morphine analgesia was enhanced, with abolition of antinociceptive tolerance (14, 15) and reduction of respiratory depression and acute constipation (16). These data suggested that whereas G protein-dependent pathway mediated analgesia, beta-arrestin-dependent signaling pathway promoted side effects. Using different strategies [high-throughput screening, structure-based virtual screening, or synthesis-driven approach (17)], new biased MOR ligands were discovered. They are mostly biased toward G proteins.

## Biased Ligands are Promising Compounds

The most notable compound, TRV-130 (Oliceridine) was discovered after a high-throughput screening by Trevena Inc. (18). It has a moderate biased toward cAMP pathway (about 3-fold) and was unable to promote MOR endocytosis according to its very weak ability to recruit beta-arrestin 2. Preclinical studies showed that TRV130 had a higher potency than morphine to induce antinociceptive responses with a reduced respiratory depression and gastrointestinal effects, suggesting a better therapeutic index as compared to morphine (19). Moreover, in the tail immersion test, no tolerance was measured after a 3 days treatment (20). Clinical studies validated these results. Indeed, in a phase 2 study comparing TRV130 with morphine in patients after abdominoplasty, the biased agonist provided not only a quicker analgesia, but was also associated with fewer side effects including hypoventilation, nausea, and vomiting (21). In a phase 3 study, TRV130 was shown to be efficient in reducing pain intensity in two surgical procedures: bunionectomy (APOLLO-1 trial) (22) and abdominoplasty (APOLLO-2 trial) (23). Analysis of side effects in the pooled cohorts of APOLLO trials showed that whereas TRV130 induced a dose dependent respiratory depression, this effect is lower than the one induced by morphine (24), and that TRV130 had a higher probability of producing analgesia rather than respiratory depression (25). These analyses also demonstrated a lower risk of experiencing nausea and vomiting in patients treated with TRV130 compared to morphine-treated patients (26). At present, TRV130 is approved by the Food and Drug Administration (FDA) since august 2020 to treat moderate to severe acute pain. Finally, TRV130 turned to be a good analgesic medication but its advantages, especially regarding side effects, toward classical opioids such as morphine is still matter of debate.

Thanks to the progress in GPCR crystal structure, Manglik and co-workers used the MOR crystal structure to virtually dock millions of compounds and they discovered PZM21 (27). PZM21 was found to be biased toward G protein pathway and active in both spinal and supra-spinal analgesia in rodents (27, 28). Interestingly, this compound seemed to induce less respiratory depression and constipation than morphine (27).

Finally, the latest notable MOR biased compounds are a series of a piperidine core structure-containing molecules (SR series) generated from a synthesis–driven approach. Some of these compounds were found to be biased, at different levels, toward G protein. They induced analgesia (measured in the hot plate and tail flick tests), showed minor respiratory depression and are brain penetrant (29). Interestingly, SR-17018 was demonstrated to be more potent and efficacious than morphine or oxycodone in a chemotherapeutic-induced neuropathy pain model and displayed a weak tolerance after a repeated administration (30). Finally, when substituted to morphine after a chronic treatment, it prevents the onset of morphine withdrawal (31).

## But They Remain MOR Ligands (With Their Side Effects)

Because they bind and activate MOR, all these biased ligands could still promote main effects attributed to MOR stimulation including reward system activation. Indeed, in rats, TRV130 displayed same effects as morphine in intracranial self-stimulation (20). In a drug-discrimination experiment conducted in rats, two biased ligands TRV130 and SR-14968 generalized to fentanyl (32), suggesting that these ligands may produce prototypic MOR agonist abuse-related effects. Using a model closer to the human behavior, intravenous self-administration procedure in rats, Austin Zamarripa and colleagues demonstrated that TRV130 was equi-potent to oxycodone (33). More recently, whereas PZM21 demonstrated a lack of effect in the conditioned place preference paradigm in two independent studies in mice (27, 28), reinforcing effects, here again comparable to those induced by oxycodone, were demonstrated using a self-administration procedure in monkeys (34), but not in rats (28). All these data from animal studies could suggest that the MOR biased compounds could have reinforcing effects in humans. Indeed, in a clinical study assessing the analgesic effects of TRV130 in healthy volunteers, Soergel and collaborators evaluated the abuse-related subjective effects with a drug effects questionnaire. They found that the dose of 3 mg, equianalgesic to 10 mg morphine, induced the same subjective opioid effects such as “high” or “liking” (35).

These data highlight the importance of the model at two level: the species and the experimental model used. Furthermore, in the same species, the choice of the strain of animals used can also have a very important impact on the results. Thus, the first results obtained by Bohn and co-workers on beta-arrestin 2 knockout animals were obtained on a mixed genetic background (beta-arrestin 2 knockout 129/SvJ backcrossed with C57BL6). Previous studies reported that the 129/SvJ mice have a higher sensitivity to morphine-induced antinociception (36), did not develop tolerance to morphine-induced analgesia (37) and were less sensitive to respiratory depression (38). When reassessing the pharmacological responses induced by opioids in beta-arrestin 2 knockout using an homogenous C57BL6 background, Koblich and colleagues did not find any difference in tolerance to analgesic effects of few opioids including morphine (39). More recently, Kliewer and co-workers reported that the knockout of beta-arrestin 2 in C57BL6 mice did not alter the effect of morphine on both respiratory depression and constipation compared to wild-type animals (40). Taken together, these data suggested that the use of 129/SvJ strain might have misled us on the role of beta-arrestin 2 in morphine effects and as a consequence it questions the G protein biased agonists as valuable strategy for safer analgesic medication. More importantly, an increase number of studies challenged the existence of the biased signaling itself (41). Evaluating the bias usually consist of measuring the ability of an agonist to stimulate two signaling pathways. At end, one will compare an amplified assay of G activation vs. an unamplified assay of beta-arrestin recruitment. Amplified assay are relatively insensitive to agonist efficacy differences. Whereas, an agonist with a low intrinsic efficacy will show a maximal effect in G protein assay, it will have a lower maximal effect in beta-arrestin assay. Using newly unamplified probes to measure signaling, several studies demonstrated that the newly biased ligands are in fact agonists with a low intrinsic efficacy relative to morphine (42–44). These recent data show that while biased ligands may have benefits over unbiased ligands, particularly in terms of the reduction of side effects, these may not be due to the biased nature of the compounds (45).

## Concluding Remarks

It took more than 30 years from the first observations of a biased signaling and the release of Oliceridine, the first biased opioid receptor compound on the market. In the last few years, some studies have challenged this concept by suggesting that the observed bias is rather the expression of the agonist low intrinsic efficacies (42) or binding kinetics (46). Therefore, the promises of having opioid ligands with virtually a lack of side effects will still take time. Rather than a revolution, the biased compounds are a hopeful evolution in the pharmacological research of analgesics. The pharmacology of GPCRs is very complex, and the simplistic view of one receptor, one signaling pathway, one pharmacological response is a far too simplistic concept, which does not reflect the complexity of life sciences. So, should we abandon opiate ligands to treat pain? Certainly not, for now, as they remain the most effective antinociceptive agents. Nevertheless, alternative research strategies should be explored further. The development of non-selective opioid ligands (agonist or mixed agonist/antagonist) could be an interesting approach. For instance, cebranopadol, a mixed MOR/DOR/KOR/NOR agonist was found to be efficient in both acute and chronic pain with a delayed development of tolerance as compared to morphine (47). This compound is actually in phase 3 trials. More recently, Ding and co-workers described the AT-121, a MOR/NOR mixed agonist with analgesic effects in non-human primates and a lack of the most frequent opioid-associated side effects such as physical dependence, abuse potential, respiratory depression, and opioid-induced hyperalgesia (48). Allosteric ligands at the MOR could be also interesting as they could be used to enhance the effects of either the exogenous orthosteric agonists when administrated together or the endogenous opioid peptides released in stress or pain condition (49). Another strategy could be the development of dual-target ligands directed to both MOR and another receptor, including for instance dopamine D3 receptor (50). Whether biased ligands, ligands with low selectivity toward different opioid receptors, or bivalent ligands, the development of an analgesic compound free of side effects and with low abuse potential seems to be a way still paved with some difficulties.

## Author Contributions

NM and FN wrote the manuscript. All authors take responsibility for final content. All authors read and approved the final manuscript.

## Conflict of Interest

The authors declare that the research was conducted in the absence of any commercial or financial relationships that could be construed as a potential conflict of interest.

## Publisher's Note

All claims expressed in this article are solely those of the authors and do not necessarily represent those of their affiliated organizations, or those of the publisher, the editors and the reviewers. Any product that may be evaluated in this article, or claim that may be made by its manufacturer, is not guaranteed or endorsed by the publisher.
